# A Comprehensive Assessment of the Nutritional Value, Antioxidant Potential, and Genetic Diversity of *Fenneropenaeus merguiensis* from Three Different Regions in China

**DOI:** 10.3390/biology13121002

**Published:** 2024-12-02

**Authors:** Yundong Li, Juan Chen, Siyao Cao, Ziyi Jiang, Song Jiang, Qibin Yang, Lishi Yang, Jianhua Huang, Jianzhi Shi, Zhenhua Ma, Falin Zhou

**Affiliations:** 1 South China Sea Fisheries Research Institute, Chinese Academy of Fishery Sciences, Key Laboratory of South China Sea Fishery Resources Exploitation and Utilization, Ministry of Agriculture and Rural Affairs, Guangzhou 510300, China; liyd2019@163.com (Y.L.); chenj29725@163.com (J.C.); cao13793933909@163.com (S.C.); tojiangsong@163.com (S.J.); yangqibin1208@163.com (Q.Y.); yangls2016@163.com (L.Y.); huangjianhua@scsfri.ac.cn (J.H.); shijianzhi1989@163.com (J.S.); zhenhua.ma@scsfri.ac.cn (Z.M.); 2 College of Fisheries and Life Sciences, Dalian Ocean University, Dalian 116023, China; 3 Key Laboratory of Efficient Utilization and Processing of Marine Fishery Resources of Hainan Province, Sanya Tropical Fisheries Research Institute, Sanya 572018, China; 4 Shenzhen Base of South China Sea Fisheries Research Institute, Chinese Academy of Fishery Sciences, Shenzhen 518108, China; 5 Keck School of Medicine, University of Southern California, 1975 Zonal Ave., Los Angeles, CA 90033, USA; ziyijian@usc.edu

**Keywords:** *Fenneropenaeus merguiensis*, nutritional composition, germplasm resources, genetic diversity

## Abstract

*Fenneropenaeus merguiensis* is one of the largest species of penaeid shrimp. It has a wide distribution of germplasm resources in the South China Sea and the southeastern coastal regions of China, yet its germplasm characteristics remain insufficiently understood. Therefore, we conducted analyses of basic nutritional components, amino acids, fatty acids, antioxidant indices, and genetic diversity in three *F. merguiensis* populations (FmRP, FmSZ, FmSY). The results showed a significant difference in ash content, with FmSZ having the highest ash content (1.77 g/100 g) (*p* < 0.05). A total of 17 amino acids were detected, and FmSZ exhibited higher concentrations of most amino acids, especially essential amino acids (*p* < 0.05). The analysis of fatty acid composition revealed significant differences between the populations, with FmSY potentially having a nutritional advantage overall (*p* < 0.05). In terms of antioxidant capacity and genetic diversity, there were no significant differences in total antioxidant capacity (T-AOC), catalase (CAT), or superoxide dismutase (SOD) among the populations (*p* > 0.05). However, genetic analysis indicated that FmSZ had the lowest inbreeding coefficient and relatively higher genetic diversity. In conclusion, among the three *F. merguiensis* populations, FmSZ has relatively higher nutritional quality and genetic diversity, and *F. merguiensis* is a high-quality aquatic product worth promoting. The analysis and characterization of various aspects of *F. merguiensis* from the three locations provide important information and data support for germplasm resources and genetic breeding efforts.

## Simple Summary

*Fenneropenaeus merguiensis*, one of the largest penaeid shrimp species, has a wide germplasm distribution along the southeastern coast of China, although its germplasm characteristics remain inadequately explored. This study conducted comprehensive analyses of basic nutritional components, amino acids, fatty acids, antioxidant indices, and genetic diversity across three *F. merguiensis* populations (FmRP, FmSZ, FmSY). The findings revealed that FmSZ had significantly higher ash, crude protein, and essential amino acid levels, indicating superior nutritional quality. FmSY showed a potential nutritional advantage due to its fatty acid profile. All populations demonstrated high levels of polyunsaturated fatty acids (DHA, EPA), along with strong antioxidant capacities. Genetic analysis showed FmSZ had the lowest inbreeding coefficient and relatively higher genetic diversity. Overall, FmSZ stands out as a high-quality candidate for aquaculture and genetic breeding, highlighting the value of *F. merguiensis* as an aquatic product with considerable potential for wider promotion and development.

## 1. Introduction

*Fenneropenaeus merguiensis* is widely distributed in the Southern Hemisphere, from East Africa to Australia, as well as in the Northern Hemisphere across Southeast Asia and the Indian Ocean [1,2,3]. In China, *F. merguiensis* is primarily distributed in the coastal areas of Guangdong, Hainan, and Guangxi. As an economically significant warm-water shrimp, it is recognized for its rapid growth, tender meat, and strong disease resistance, which make it a valuable species for commercial aquaculture [4]. Despite its high commercial value and popularity as a seafood choice, the lack of a stable, artificially bred seed supply system has hindered the development of large-scale farming. Currently, the aquaculture industry faces challenges in maintaining a consistent supply of juveniles, limiting the expansion of farming operations and the species’ full potential in the market. Nevertheless, *F. merguiensis* remains a focus of research due to its promising growth characteristics and overall high-quality meat.

As the farming industry for *F. merguiensis* has developed, artificial breeding of the species has increasingly gained importance. Currently, much of the global research on shrimp germplasm resources focuses on species such as *Litopenaeus vannamei* [5] and *Penaeus monodon* [6]. *F. merguiensis* has great potential as a cultured species, but research on its germplasm resources is limited. The studies that exist mainly concentrate on its genetic diversity [1,7], growth performance [8], and disease resistance [4]. A fatty acid composition analysis of *F. merguiensis* from Pakistan identified five dominant fatty acids in its muscle: docosahexaenoic acid, eicosapentaenoic acid, oleic acid, palmitic acid, and stearic acid [9]. When assessing the impact of varying concentrations of Enteromorpha polysaccharides (EPS) on the growth of *F. merguiensis*, the results indicated that incorporating 1 g/kg of EPS into the diet notably boosted final body mass, weight gain, average daily growth rate, and specific growth rate, thereby enhancing overall growth performance. Moreover, this supplementation significantly increased the activity of various antioxidant enzymes within the hemolymph [8]. The optimal growth performance of *F. merguiensis* was observed with 35% protein content and an energy-to-protein ratio of 8.5 kcal/g, while the highest feed consumption, energy digestibility, and survival rate occurred with 40% protein and a ratio of 9.5 kcal/g. Regression analysis suggested that the ideal protein content is 34.4%, with an energy-to-protein ratio of 8.5 kcal/g [10]. Genetic diversity studies on *F. merguiensis* predominantly use microsatellite markers to characterize the genetic traits of different populations [1,11].

Currently, research on *F. merguiensis* is mainly focused on fundamental areas. Although some preliminary achievements have been made in germplasm resources and genetic diversity, studies in these fields remain relatively scarce. Especially within the context of China, comprehensive studies focusing on distinct regional demographics are particularly scarce. In order to fill the void in this area of research, we have chosen key agricultural sites across China (Raoping, Shenzhen, and Sanya) to systematically assess the germplasm resources of *F. merguiensis*, evaluate its nutritional value, and build a theoretical foundation for establishing a germplasm resource bank. Furthermore, we carried out an in-depth analysis of the genetic diversity of *F. merguiensis* across the three farming sites. This comprehensive study is designed to establish a robust basis for exploring the germplasm resources of *F. merguiensis* and to inform scientific management of shrimp resources in these agricultural regions. This will not only help promote the conservation and sustainable use of the species but also provide valuable data support for future genetic research and aquaculture practices.

## 2. Materials and Methods

### 2.1. Sample Collection

**Figure 1:**
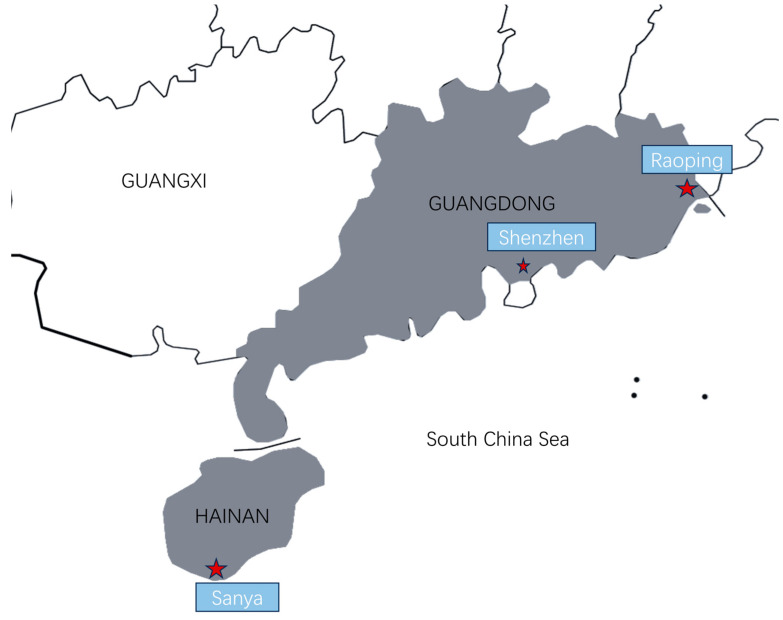
Geographical representation of the survey location.

Based on the legal and regulatory provisions, as well as the diversity of *F. merguiensis* farming locations in China, we selected three representative farming sites for sample collection: Raoping, Sanya, and Shenzhen (Table 1, Figure 1). We employed calibrated equipment like pH meters, dissolved oxygen meters, and salinity meters, along with chemical test kits utilizing colorimetric techniques (Merck KGaA, Darmstadt, Germany), to track water quality indicators including salinity, ammonia, nitrite, pH levels, dissolved oxygen, and nitrate levels. The results indicated that these parameters were largely uniform across various populations, suggesting that the farming environments were broadly similar across the different regions. Specifically, the water temperature at each sampling site was maintained between 27.4 and 28.5 °C, and the pH was kept around 8.8.

At each selected location, we randomly selected 30 live shrimps and their body length and weight were recorded. Subsequently, 1 g of muscle tissue was extracted from each shrimp, which was then placed into centrifuge tubes and preserved at −80 °C. For regular nutritional analysis, including amino acids, fatty acids, and enzymes, three random shrimps were chosen per parameter. The remaining samples were reserved for genetic analysis to assess germplasm traits.

### 2.2. General Nutrition Analysis

At every sampling location, three randomly chosen, healthy *F. merguiensis* individuals in the intermolt phase were selected to collect muscle tissue for in-depth analysis. The determination of moisture content was based on the National Food Safety Standard, specifically the “Determination of Moisture in Food” using the direct drying method as outlined in GB 5009.3-2016 [12]. The protein content was quantified using the Kjeldahl method as detailed in GB 5009.5-2016 [13], ensuring the precision of the measurement. The fat content was evaluated based on the Soxhlet extraction method described in GB/T 5009.6-2016 [14]. Furthermore, the ash content was analyzed in accordance with the Ashing method specified in GB 5006.4-2016 [15], and the total sugar content was measured using the Phenol-sulfuric acid colorimetric method specified in GB/T 15672-2009 [16], which is the standard for “Determination of Total Saccharide in Edible Mushroom.” These methods ensured that our nutritional analysis was both thorough and precise.

### 2.3. Physiological Analysis

The composition of amino acids in the hydrolyzed samples was assessed according to the National Food Safety Standards, specifically adhering to the “Determination of Amino Acids in Food” protocol using high-performance liquid chromatography as detailed in GB 5009.124-2016 [17]. In addition, the fatty acid profile of the samples was ascertained following the “Determination of Fatty Acids in Food” using the External standard method, as per GB 5009.168-2016 [18]. For the assessment of antioxidant enzyme activities, superoxide dismutase (SOD) activity was measured using the WST-8 kit (A001-1-2) procured from Nanjing Jiancheng Bioengineering Institute. The assessment of total antioxidant capacity (T-AOC) was conducted using a suitable assay kit (A015-1), and catalase (CAT) activity was determined using a kit (A007-1-1) also supplied by Nanjing Jiancheng Bioengineering. Throughout the experimental process, strict adherence to the protocols was maintained. Samples were meticulously prepared, and reagents were accurately dispensed to initiate the enzymatic reactions. Reactions were halted at the designated time intervals to ensure the accurate detection of reaction products and the precise measurement of enzyme activities, with all findings meticulously documented.

### 2.4. Genetic Diversity

In this research, muscle samples from randomly selected live shrimps across various regions underwent genetic diversity analysis. The genomic DNA quality was verified, and ultrasonication was employed to fragment the DNA. Purification and end-repair processes were conducted, followed by adenylation and adapter ligation. Agarose gel electrophoresis was used to select fragments of the appropriate size, which were then amplified by PCR to generate sequencing libraries. Following quality assessment, these libraries were subjected to sequencing on the Illumina sequencing platform. Fastp software (0.23.2) processed the raw data, filtering out reads with excessive adapter sequences, low-quality bases, or high N content, resulting in clean and high-quality data. The purified data were subsequently aligned using Sentieon version 201711.03 [19] and the advanced Burrows-Wheeler Aligner (BWA) MEM algorithm developed by the Wellcome Trust Sanger Institute in Cambridge, UK. This approach not only expedited the alignment process but also ensured that the alignments maintained a high level of precision [20,21]. Genetic variants were identified using Sentieon and HaplotypeCaller in joint-genotyping mode to generate gVCF files and detect SNPs across populations. High-precision SNP detection was ensured by applying quality control based on GATK’s filtering criteria. SNPs with a detection frequency over 60% were selected using GATK’s SelectVariants and VariantFiltration tools. Genetic diversity metrics including SNP frequency, nucleotide diversity, inbreeding coefficient, polymorphic information content, and observed heterozygosity were determined using VCFtools and tailored scripts. SNP detection in BAM files was performed with GATK, and the F_ST_ index was used to measure genetic differentiation among populations. To improve data quality, low-quality reads were removed using SOAPnuke software (https://github.com/BGI-flexlab/SOAPnuke, 2.X version, accessed by 29 October 2024) [22]. The genomic sequencing and bioinformatics analyses conducted have laid a robust foundation and provided pivotal insights into the assessment and understanding of genetic variability within the shrimp samples.

### 2.5. Data Analysis

In this research, a one-way ANOVA test was conducted utilizing SPSS 16.0 to assess statistical significance, with the threshold for significance defined as *p* < 0.05. Outcomes exceeding this threshold (*p* > 0.05) were deemed statistically insignificant. When the ANOVA indicated significance, Duncan’s test was employed to discern specific differences among the groups. When the ANOVA indicated significance, Duncan’s test was employed to discern specific differences among the groups. Data are expressed as mean ± standard deviation, ensuring the precision and reliability of the outcomes. Genetic variation was measured using various indices in our analysis, including SNP density = Number of SNPs/Genome size (Kb), PIC = 1 − ∑pi^2^ − ∑∑2pi^2^pj^2^, Ho = het/(het + hom), π = n/(n − 1) ∑pipjπij, F = (Ho − He)/(n − He), F_ST_ = (HT − HS)/HT.

## 3. Results

### 3.1. Analysis of General Nutritional Composition

In the nutritional analysis of *F. merguiensis* muscle tissue, no significant differences (*p* > 0.05) were found in moisture, crude protein, crude fat, and total sugar among the three cultured populations. However, there were notable differences in ash content (*p* < 0.05, refer to Table 2). The ash content varied from 1.37 to 1.77 g/100 g, with FmSZ recording the maximum at 1.77 g/100 g. Regarding moisture, FmRP exhibited the highest content at 75.43 g/100 g, and FmSZ the lowest at 73.87 g/100 g. The crude fat content spanned from 0.90 to 1.03 g/100 g. Interestingly, FmRP showed the lowest levels in crude protein and total sugar, at 20.83 g/100 g and 0.32%, respectively. Conversely, FmSY had the highest crude protein content at 21.70 g/100 g, and FmSZ had the highest total sugar content at 0.40%.

### 3.2. Amino Acids Results of F. merguiensis

**Figure 2:**
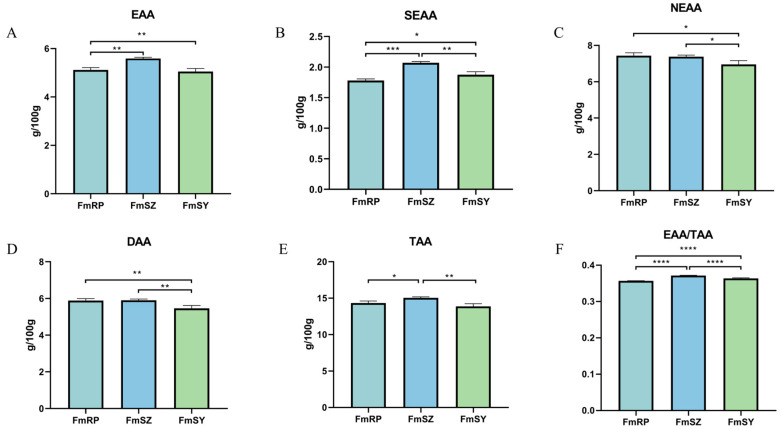
Muscle amino acid composition and content of three groups of *F. merguiensis*. (**A**): EAA: essential amino acids. (**B**): SEAA: total semi-essential amino acids. (**C**): NEAA: total non-essential amino acids. (**D**): DAA: delicious amino acids. (**E**): TAA: total amino acids. (**F**): EAA/TAA: essential amino acids/total amino acids. Different number of symbols between treatments indicate significant differences (* 0.01 < *p* < 0.05; ** *p* < 0.01, *** *p* < 0.001, **** *p* < 0.0001).

Following the evaluation of amino acid profiles in FmRP, FmSZ, and FmSY, we identified a total of 17 amino acids, comprising 7 essential, 2 conditionally essential, and 8 non-essential amino acids (Table 3, Figure 2). Most amino acid types exhibited significant differences (*p* < 0.05). Overall, FmRP and FmSY showed a trend of no significant differences (*p* > 0.05) in most amino acid types, while FmSZ exhibited significant differences (*p* < 0.05) compared to the other two populations. Among all detected amino acids, glutamic acid had the highest content, ranging from 2.07 to 2.33 g/100 g, while cysteine had the lowest content, ranging from 0.10 to 0.14 g/100 g. It is worth mentioning that certain amino acids, including aspartic acid (Asp), threonine (Thr), serine (Ser), glutamic acid (Glu), glycine (Gly), alanine (Ala), valine (Val), methionine (Met), isoleucine (Ile), phenylalanine (Phe), lysine (Lys), histidine (His), and arginine (Arg), were particularly abundant in the FmSZ group. Conversely, cysteine (Cys), leucine (Leu), and tyrosine (Tyr) were found to be more prevalent in the FmRP group. Overall, FmSY had lower levels of most amino acids compared to FmRP and FmSZ, while FmSZ exhibited the highest values for most amino acids. Additionally, the FmSZ samples showed the highest values in EAA (essential amino acids), SEAA (semi-essential amino acids), DAA (non-essential amino acids), TAA (total amino acids), and EAA/TAA, with significant differences among the groups.

### 3.3. Results of Fatty Acids Composition

**Figure 3:**
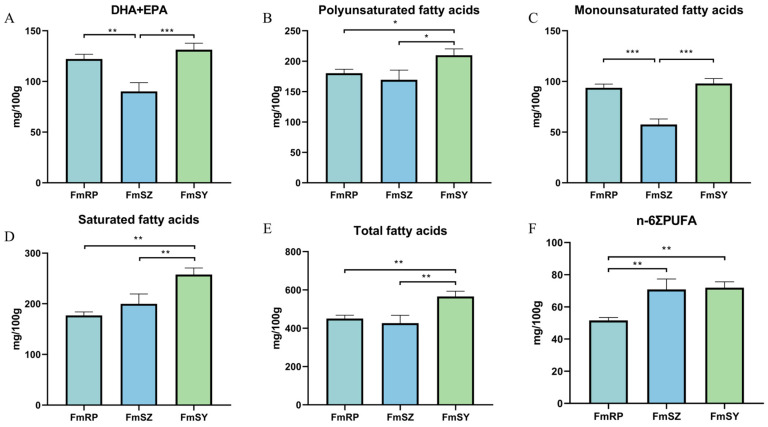
Muscle fatty acid composition and content of three groups of *F. merguiensis*. (**A**): the content of DHA + EPA. (**B**): the content of polyunsaturated fatty acids. (**C**): the content of monounsaturated fatty acid. (**D**): the content of saturated fatty acid. (**E**): the content of total fatty acid. (**F**): the content of n-6ΣPUFA. Different number of symbols between treatments indicate significant differences (* 0.01 < *p* < 0.05; ** *p* < 0.01, *** *p* < 0.001).

After a detailed analysis of the fatty acid composition in the three populations (FmRP, FmSZ, and FmSY), we revealed significant differences in the content of various fatty acids (*p* < 0.05) (Table 4, Figure 3). Among the detected fatty acids, the content of C18:0 was the highest, ranging from 75.23 to 118.20 mg/100 g, while C20:2 had the lowest content, ranging from 3.70 to 5.23 mg/100 g. Notably, the highest content of C15:0 in FmSY was 8.50 mg/100 g, whereas the lowest content of C16:0 in FmSY was 66.43 mg/100 g. C16:1 and C17:0 had relatively low levels in FmSZ, while C18:1n9c was lower in FmSZ but higher in FmRP at 72.20 mg/100 g. The contents of C18:2n6c and C20:2 were relatively similar across the three samples. C22:0 had the highest content in FmRP and the lowest in FmSY, further emphasizing the differences in fatty acid composition among the populations. The polyunsaturated fatty acids C20:4n6 and C22:1n9 were higher in FmSZ and relatively lower in FmRP, which may reflect FmSZ’s advantage in these health-related fatty acids. Omega-3 polyunsaturated fatty acids C20:5n3 (EPA) and C22:6n3 (DHA) were higher in FmSY and lower in FmSZ. Moreover, FmSY exhibited the highest values in the total of DHA and EPA, polyunsaturated fatty acids, monounsaturated fatty acids, saturated fatty acids, total fatty acid content, and n-6 polyunsaturated fatty acids, indicating that FmSY may have an advantage in the overall nutritional value of fatty acids. These results offer a comprehensive insight into the fatty acid profiles of the three populations, establishing a foundation for subsequent studies on nutritional value and health benefits of food.

### 3.4. Oxidative Stress Indicators of F. merguiensis

**Figure 4:**
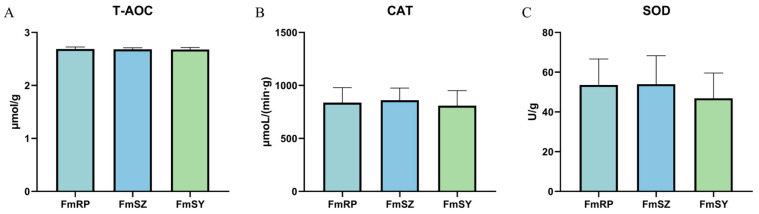
Muscle physiological and biochemical indicators composition and content of three groups of *F. merguiensis*. (**A**): The activity of T-AOC. (**B**): The activity of SOD. (**C**): The activity of CAT. Mean ± standard deviation (*n* = 3).

After analyzing the physiological and biochemical indices of the three populations of *F. merguiensis* (FmRP, FmSZ, and FmSY), we observed no significant differences among the groups in total antioxidant capacity (T-AOC), catalase (CAT), and superoxide dismutase (SOD) (*p* > 0.05) (Figure 4). The values for T-AOC ranged from 2.678 to 2.689 μmol/g, CAT values ranged from 809.0 to 861.0 μmoL/(min·g), and SOD values ranged from 46.85 to 53.98 U/g. Among these, FmSZ exhibited the highest values, while FmSY had the lowest.

### 3.5. Analysis of Genetic Diversity

When evaluating the genetic variability across the three groups, the average SNP density ranged from 1.066 to 1.081, while the values for nucleotide diversity (π) were similar, approximately between 2.18 × 10^4^ and 2.22 × 10^4^. The inbreeding coefficient varied between 12.9% and 15.9%, with FmSZ exhibiting the lowest inbreeding coefficient, indicating the least likelihood of inbreeding. The average value of polymorphic information content (PIC) was around 0.125, reflecting the diversity of genetic markers. The average observed heterozygosity (Ho) ranged from 0.116 to 0.121, which indicates the frequency of heterozygotes in the population (Table 5). Finally, the genetic differentiation index (F_ST_) demonstrated the genetic differences between populations (Table 6), with the lowest F_ST_ value of 0.002 between FmSZ and FmSY, suggesting minimal genetic differentiation between these two populations. In contrast, the F_ST_ value between FmRP and FmSZ was 0.004, and the value between FmRP and FmSY was also 0.004, indicating slightly greater genetic differences between these two groups.

## 4. Discussion

### 4.1. Comparative Analysis of Basic Nutritional Components Across F. merguiensis Populations

Aquatic creatures serve as premium dietary sources, prized for their rich protein, minimal fat, and low-calorie attributes [23]. The nutritional value of aquatic animals’ muscle tissue is primarily determined by the combined presence of key constituents, namely moisture, ash, crude protein, and crude fat. The muscle’s water content, being its most prevalent constituent, plays a crucial role in influencing its tenderness and flavor, and greatly affects the muscle’s water-holding capacity [24]. Ash, encompassing a variety of minerals, is present in small quantities but is essential for sustaining the life processes and well-being of both animals and humans. Crude protein, reflecting the muscle’s total protein content in aquatic animals, is a key nutritional metric, vital for their growth, development, and tissue repair [25]. In recent decades, with the rapid improvement in socio-economic levels, the demand for high-quality aquatic food has continuously increased, and the aquaculture industry has experienced a rapid expansion. Therefore, a comprehensive assessment of these components is essential for evaluating and ensuring the nutritional value and food quality of aquatic products. When analyzing the conventional nutritional components of three populations of *F. merguiensis*, it was found that there were significant differences in the ash content among the three farmed populations (*p* < 0.05). The ash content of the three populations ranged from 1.37 to 1.77 g/100 g, with the highest ash content in FmSZ, reaching 1.77 g/100 g. Compared with *Penaeus chinensis* (1.69 g/100 g) and *L. vannamei* (1.52 g/100 g) [26], FmSZ has relatively higher ash content. The content of crude protein ranged from 20.83 to 21.97 g/100 g, which is higher than that of *L. vannamei* (15.09 g/100 g), *Penaeus japonicus* (14.60 g/100 g), *P. monodon* (13.29 g/100 g), and *Macrobrachium rosenbergii* (12.33 g/100 g) [27], among which, the crude protein of FmSZ and FmSY is slightly higher than that of FmRP. The content of crude fat is also lower than that of *p. japonicus* (2.09 g/100 g) [28]. In general, the three populations of *F. merguiensis* are a type of food with high protein and low fat, among which, FmSZ has relatively higher nutritional value.

### 4.2. Comparative Analysis of Amino Acids Across F. merguiensis Populations

Amino acids play a crucial role in the muscle of aquatic animals, participating in a variety of physiological functions and metabolic processes [29,30]. They are essential for the growth, development, reproduction, and tissue repair of aquatic animals [31]. In addition, amino acids are also related to the water retention, structural maintenance, and motor capacity of muscles [32]. Based on the FAO/WHO’s recommended model, the EAA/TAA ratio for high-quality proteins should ideally be 40%. Although the ratio for the three populations is slightly below this benchmark, it remains relatively close, suggesting a fairly balanced amino acid profile and classifying their protein as high-quality for human consumption. Moreover, it can be seen from the figure that the EAA of the three populations is less than 6.0 g/100 g, which is lower than that of *Haliotis japonica* (6.59 g/100 g) [33]. In addition, the composition and content of DAA can significantly influence the palatability of animal protein [34]. The results of this study found that among the three populations of *F. merguiensis*, FmSZ showed the highest values in most amino acids, and also showed the highest values in EAA, SEAA, DAA, TAA, and EAA/TAA, with significant differences between the populations. In summary, the amino acid level of *F. merguiensis* is at a medium level among shrimps, and among the three populations, FmSZ was consistently present at a notably elevated concentration, suggesting that it possesses superior nutritional benefits and a palatable flavor.

### 4.3. Comparative Analysis of Fatty Acids Across F. merguiensis Populations

Fatty acids play a vital role in aquatic animals, including providing energy, constructing cell membranes, participating in metabolic regulation, and maintaining normal growth and reproductive functions [35,36]. Furthermore, the fatty acid profile in muscle tissue is a critical determinant of both quality and taste [37]. Among the fatty acids, polyunsaturated fatty acids (PUFA), notably eicosapentaenoic acid (EPA) and docosahexaenoic acid (DHA), serve as primary precursors for the synthesis of bioactive anti-inflammatory compounds and are essential for brain development and the prevention of inflammatory cardiovascular diseases [38,39,40]. In this study, the total content of ΣPUFA in the three populations of *F. merguiensis* ranged from 169.5 to 209.8 mg/100 g, and ΣMUFA ranged from 57.47 to 97.97 mg/100 g, which is higher than that of *P. chinensis* (42.58 mg/100 g, 18.85 mg/100 g) [26] and *L. vannamei* (38.11–44.88 mg/100 g, 14.79–17.67 mg/100 g) [41]. All three populations of *F. merguiensis* showed a trend of ΣPUFA > ΣMUFA, which is consistent with the trend of *P. monodon* [6] and *L. vannamei* [5]. Moreover, the content of DHA + EPA in the three populations was 90.20-131.4 mg/100 g, which is higher than that of *L. vannamei* (103.30–115.10 mg/100 g) [5] and *P. japonicus* (11.58 mg/100 g) [27]. Most fatty acids showed a significant difference (*p* < 0.05), indicating differences in diet and environmental conditions among different populations. The WHO advises that the intake of saturated fats should be reduced to below 10% of total energy consumption. With the saturated fat content in *F. merguiensis* not exceeding 300 mg/100 g, it demonstrates that the saturated fat intake from *F. merguiensis* is well below this 10% benchmark. Consequently, *F. merguiensis* can be classified as an excellent source of nutrition, adhering to the healthy dietary standards recommended by the WHO. In summary, in terms of fatty acids, *F. merguiensis* has a high content of fatty acids and is of high nutritional value. Among the three populations, FmSZ has relatively higher overall values and more significant differences compared to the other two populations; thus, FmSZ has a relatively higher nutritional value.

### 4.4. Comparative Analysis of Physiological Indicators Across F. merguiensis Populations

In this study, no substantial variations were observed in the physiological indicators among the three populations of *F. merguiensis*. Antioxidant enzyme activity is a crucial indicator of an organism’s antioxidant capacity and is often used to assess its response to oxidative stress [42]. Aquatic organisms rely on the antioxidant enzyme system to neutralize potentially harmful reactive oxygen species (ROS), maintaining cellular integrity and physiological functions, and maintaining the redox balance within cells [43,44]. T-AOC, serving as an encompassing indicator of antioxidant potential, mirrors the collective antioxidant profile of an organism [45]. An elevated T-AOC level generally indicates a potent antioxidant mechanism, which is beneficial for shielding cells against oxidative stress and sustaining overall well-being [46]. SOD, an essential antioxidant enzyme, neutralizes superoxide anion radicals, protecting the body against oxidative stress [43]. CAT efficiently transforms hydrogen peroxide into water and oxygen, protecting cells from oxidative stress [47]. Therefore, the higher the activity of these antioxidant enzymes, the stronger the antioxidant and immune capabilities of the organism. In our research, the T-AOC levels in the three *F. merguiensis* populations ranged from 2.68 to 2.69 μmol/g, surpassing the T-AOC activity found in *P. clarkii*, which was recorded at 2.57 U/mg protein [48]; the SOD activity in the three populations of *F. merguiensis* was between 46.85 and 53.98 U/g, which is greater than the SOD activity observed in *P. monodon*, ranging from 39.65 to 47.29 U/g [6], indicating that the three different populations of *F. merguiensis* all showed higher antioxidant enzyme activity, among which FmSZ showed higher activity in various antioxidant enzymes, thus FmSZ has higher antioxidant enzyme activity and antioxidant capacity.

### 4.5. Comparative Analysis of Genetic Diversity Across F. merguiensis Populations

Genetic diversity is essential for a species’ ability to adapt to dynamic environments, supporting its survival and evolutionary potential. Higher genetic diversity is beneficial for enhancing a species’ environmental adaptability, growth, and disease resistance [49,50]. Parameters like Polymorphic Information Content (PIC) are vital for evaluating the genetic diversity within populations [51]. The PIC value reflects the probability that a marker will exhibit different allelic genotypes when two individuals are randomly selected from a population. A PIC value greater than 0.5 is considered highly polymorphic, values between 0.25 and 0.5 are considered moderately polymorphic, and values below 0.25 are classified as low polymorphism [52]. SNP density reflects how frequently SNP sites occur across the genome, with a higher density generally associated with increased genetic diversity [53]. Nucleotide diversity evaluates the differences among nucleotide sites within a defined genetic region, serving as a vital indicator of genetic diversity among populations [54]. The F_ST_ index evaluates the level of genetic differentiation among populations, with elevated F_ST_ values suggesting substantial genetic separation [55]. In the genetic diversity analysis of the FmRP, FmSZ, and FmSY populations, it was found that their SNP density ranged from 1.066 to 1.081, nucleotide diversity (π) values were between 2.18 × 10^−4^ and 2.22 × 10^−4^, and PIC was within the range of 0.124–0.125, indicating low genetic diversity. Additionally, the inbreeding coefficient (F_ST_) of these three populations ranged from 0.002 to 0.004, also showing low values. These findings imply that the genetic variability within the three *F. merguiensis* populations is low, and there is no significant genetic differentiation among them. Among these populations, FmSZ exhibits a comparatively higher level of genetic diversity, whereas FmRP and the other population show a relatively greater extent of genetic differentiation.

## 5. Conclusions

This study analyzed the nutritional components, amino acid composition, fatty acid profile, antioxidant capacity, and genetic diversity of *F. merguiensis*. The results indicate that *F. merguiensis* is a high-quality aquatic food, characterized by high protein and low fat content. The FmSZ population stands out with significantly higher levels of ash, crude protein, and optimal amino acids, enhancing its nutritional value. All populations exhibited high levels of polyunsaturated fatty acids, particularly DHA and EPA, suggesting potential health benefits. Antioxidant assays demonstrated strong resistance to oxidative stress, with FmSZ showing superior antioxidant enzyme activity. While genetic diversity is relatively low, the overall performance of the FmSZ population underscores its significance for aquaculture and food security. Among the three *F. merguiensis* populations, FmSZ displays relatively higher nutritional quality and genetic diversity. This research positions *F. merguiensis* as a high-quality aquatic species with substantial potential for broader promotion and development. Specifically, the FmSZ population is identified as having notable potential for further advancement in the aquaculture industry, offering a nutritious and healthy option for consumers. Future research should focus on enhancing the genetic diversity of *F. merguiensis*, exploring the molecular mechanisms behind its nutritional and antioxidant properties, assessing environmental impacts, and investigating its food safety and meat quality to support its industrial applications.

## Figures and Tables

**Table 1:** Fundamental details regarding the survey location.

Population	Body Length (mm)	Weight (g)	Category	Location
Raoping (FmRP)	179.8 ± 8.2	80.2 ± 7.9	Representative enterprise	23°66′ N, 117°00′ E
Shenzhen (FmSZ)	175.5 ± 4.3	80.1 ± 2.2	Genetic breeding center	22°49′ N, 114°59′ E
Sanya (FmSY)	172.00 ± 12.00	73.60 ± 17.82	Genetic breeding center	18°30′ N, 110°10′ E

Body length and weight are presented as mean ± standard deviation (*n* = 30).

**Table 2:** Nutritional composition and content in the muscle tissue across three populations of *F. merguiensis*.

Nutritional Components	FmRP	FmSZ	FmSY
Ash (g/100 g)	1.63 ± 0.06 ^a^	1.77 ± 0.06 ^a^	1.37 ± 0.06 ^b^
Moisture (g/100 g)	75.43 ± 0.25 ^a^	73.87 ± 1.02 ^a^	74.17 ± 0.55 ^a^
Crude fat (g/100 g)	1.00 ± 0.10 ^a^	1.03 ± 0.15 ^a^	0.90 ± 0.10 ^a^
Crude protein (g/100 g)	20.83 ± 0.46 ^a^	21.70 ± 1.71 ^a^	21.97 ± 1.31 ^a^
Total sugar (%)	0.32 ± 0.03 ^a^	0.40 ± 0.08 ^a^	0.35 ± 0.03 ^a^

mean ± standard deviation (*n* = 3). Superscript letters (a, b) within the same row denote statistically significant differences between groups. Values sharing the same letter are not significantly different (*p* > 0.05).

**Table 3:** Amino acid profile and concentration in the muscle tissues of the three *F. merguiensis* groups.

Amino Acids (g/100 g)	FmRP	FmSZ	FmSY
Aspartic acid ^@^	1.42 ± 0.03 ^a^	1.54 ± 0.02 ^b^	1.36 ± 0.04 ^a^
Threonine *	0.55 ± 0.01 ^a^	0.60 ± 0.01 ^b^	0.55 ± 0.02 ^a^
Serine	0.46 ± 0.01 ^ab^	0.48 ± 0.01 ^a^	0.45 ± 0.02 ^b^
Glutamic acid ^@^	2.07 ± 0.04 ^a^	2.33 ± 0.03 ^b^	2.10 ± 0.07 ^a^
Glycine ^@^	1.53 ± 0.03 ^a^	1.09 ± 0.01 ^b^	1.08 ± 0.03 ^b^
Alanine ^@^	0.87 ± 0.02 ^a^	0.94 ± 0.01 ^b^	0.91 ± 0.02 ^b^
Cystine	0.14 ± 0.01 ^a^	0.13 ± 0.02 ^ab^	0.10 ± 0.01 ^b^
Valine *	0.67 ± 0.01 ^a^	0.74 ± 0.01 ^b^	0.67 ± 0.02 ^a^
Methionine *	0.36 ± 0.01 ^a^	0.39 ± 0.02 ^b^	0.35 ± 0.01 ^a^
Isoleucine *	0.61 ± 0.01 ^a^	0.67 ± 0.01 ^b^	0.60 ± 0.02 ^a^
Leucine *	1.33 ± 0.02 ^a^	1.24 ± 0.02 ^b^	1.11 ± 0.03 ^a^
Tyrosine	0.29 ± 0.01 ^a^	0.25 ± 0.00 ^b^	0.24 ± 0.01 ^c^
Phenylalanine *	0.57 ± 0.01 ^a^	0.63 ± 0.01 ^b^	0.56 ± 0.01 ^a^
Lysine *	1.22 ± 0.03 ^a^	1.33 ± 0.01 ^b^	1.22 ± 0.03 ^a^
Histidine ^&^	0.27 ± 0.01 ^a^	0.31 ± 0.00 ^b^	0.28 ± 0.01 ^a^
Arginine ^&^	1.51 ± 0.02 ^a^	1.76 ± 0.02 ^b^	1.60 ± 0.05 ^c^
Proline	0.67 ± 0.03 ^ab^	0.62 ± 0.01 ^a^	0.71 ± 0.02 ^b^

Amino acid levels are presented as mean ± SD (*n* = 3). Distinct letters denote significant differences (*p* < 0.05) among treatments. ^@^ Delicious amino acids. * Essential amino acids. ^&^ Semi-essential amino acids.

**Table 4:** Muscle fatty acid composition and content of three groups of *F. merguiensis* (mg/100 g).

Fatty Acid	FmRP	FmSZ	FmSY
C15:0	4.37 ± 0.15 ^a^	5.57 ± 0.57 ^b^	8.50 ± 0.40 ^c^
C16:0	69.80 ± 2.82 ^a^	66.43 ± 6.44 ^b^	99.97 ± 4.96 ^b^
C16:1	21.60 ± 0.92 ^a^	16.33 ± 1.60 ^b^	31.40 ± 1.60 ^c^
C17:0	12.43 ± 0.50 ^a^	16.67 ± 1.55 ^b^	19.97 ± 1.05 ^c^
C18:0	75.23 ± 2.86 ^a^	96.63 ± 9.42 ^b^	118.60 ± 6.06 ^c^
C18:1n9c	72.20 ± 2.72 ^a^	41.13 ± 3.96 ^b^	66.57 ± 3.37 ^a^
C18:2n6c	6.90 ± 0.26 ^a^	7.63 ± 0.78 ^a^	7.57 ± 0.25 ^a^
C20:2	4.43 ± 0.12 ^ab^	3.70 ± 0.10 ^a^	5.23 ± 0.59 ^b^
C22:0	15.13 ± 0.57 ^a^	14.63 ± 1.50 ^a^	10.60 ± 0.50 ^b^
C20:4n6	40.27 ± 1.43 ^a^	59.47 ± 5.64 ^b^	59.17 ± 2.86 ^b^
C22:1n9	6.33 ± 0.21 ^a^	8.47 ± 0.76 ^b^	6.50 ± 0.40 ^a^
C20:5n3 (EPA)	63.57 ± 2.51 ^a^	41.77 ± 4.01 ^b^	67.73 ± 3.35 ^a^
C22:6n3 (DHA)	58.67 ± 2.05 ^a^	48.43 ± 4.58 ^b^	63.63 ± 3.01 ^a^

Mean fatty acids ± standard deviation (*n* = 3). Different letters between treatments indicate significant differences (*p* < 0.05). C15:0—Pentadecanoic acid. C16:0—Palmitic acid. C16:1—Palmitoleic acid. C17:0—Heptadecanoic acid. C18:0—Stearic acid. C18:1n9c—Oleic acid. C18:2n6c—Linoleic acid. C20:2—Eicosadienoic acid. C22:0—Behenic acid. C20:4n6—Arachidonic acid. C22:1n9—Erucic acid. C20:5n3 (EPA)—Eicosapentaenoic acid. C22:6n3 (DHA)—Docosahexaenoic acid.

**Table 5:** Statistics of genetic diversity parameters of three populations of *F. merguiensis*.

Population	SNP Density (SNP/Kb)	Nucleotide Diversity (π)	Polymorphism Information Content (PIC)	Observed Heterozygosity (Ho)	Inbreeding Coefficient (F_HOM_)
FmRP	1.066	2.18 × 10^−4^ ± 3.41 × 10^−4^	0.125 ± 0.114	0.116 ± 0.151	1.59 × 10^−1^ ± 4.90 × 10^−2^
FmSZ	1.081	2.22 × 10^−4^ ± 3.47 × 10^−4^	0.125 ± 0.113	0.121 ± 0.155	1.29 × 10^−1^ ± 9.71 × 10^−1^
FmSY	1.076	2.20 × 10^−4^ ± 3.44 × 10^−4^	0.124 ± 0.114	0.117 ± 0.152	1.47 × 10^−1^ ± 3.06 × 10^−2^

**Table 6:** Genetic differentiation index (F_ST_) statistics of three populations of *F. merguiensis*.

Population-1	Population-2	F_ST_
FmRP	FmSZ	0.004
FmRP	FmSY	0.004
FmSZ	FmSY	0.002

## Data Availability

Data are contained within the article.

## References

[1] Aziz D., Siraj S.S., Arshad A. (2020). Genetic Diversity of Banana Prawns *Fenneropenaeus merguiensis* in Malaysian Waters Using Microsatellite Markers. J. Environ. Biol..

[2] Nguyen N.H., Quinn J., Powell D., Elizur A., Thoa N.P., Nocillado J., Lamont R., Remilton C., Knibb W. (2014). Heritability for Body Colour and Its Genetic Association with Morphometric Traits in Banana Shrimp (*Fenneropenaeus merguiensis*). BMC Genet..

[3] Vance D.J., Rothlisberg P.C., Sheppard C. (2020). Chapter One—The Biology and Ecology of the Banana Prawns: Penaeus Merguiensis de Man and P. Indicus H. Milne Edwards. Advances in Marine Biology.

[4] Nguyen N.H., Phuthaworn C., Knibb W. (2020). Genomic Prediction for Disease Resistance to Hepatopancreatic Parvovirus and Growth, Carcass and Quality Traits in Banana Shrimp *Fenneropenaeus merguiensis*. Genomics.

[5] Li Y., Cao S., Jiang S., Huang J., Yang Q., Jiang S., Yang L., Zhou F. (2024). Comparative Study of Nutritional Composition, Physiological Indicators, and Genetic Diversity in Litopenaeus Vannamei from Different Aquaculture Populations. Biology.

[6] Li Y., Chen J., Jiang S., Huang J., Jiang S., Yang Q., Yang L., Shi J., Zhou F. (2024). A Comprehensive Study on Nutritional Quality, Physiological Enzyme Activity and Genetic Diversity in Six Populations of *Penaeus monodon*. Aquacult Int..

[7] Prasertlux S., Khamnamtong B., Wisuntorn E., Soonsan P., Janpoom S., Tang S., Rongmung P., Ratdee O., Ninwichian P., Sakamoto T. (2024). Genetic Diversity and Population Differentiation of Wild and Domesticated Banana Shrimp *Fenneropenaeus merguiensis*: Applications for Development of Its Breeding Program. Reg. Stud. Mar. Sci..

[8] Liu W.-C., Zhou S.-H., Balasubramanian B., Zeng F.-Y., Sun C.-B., Pang H.-Y. (2020). Dietary Seaweed (Enteromorpha) Polysaccharides Improves Growth Performance Involved in Regulation of Immune Responses, Intestinal Morphology and Microbial Community in Banana Shrimp *Fenneropenaeus merguiensis*. Fish Shellfish. Immunol..

[9] Fatima H., Ayub Z., Siddiqui G., Ali S.A. (2012). Fatty Acid Composition of Two Candidate Species of Aquaculture, *Fenneropenaeus merguiensis* and *F. penicillatus* (Crustacea: Decapoda) in Pakistan. Pak. J. Zool..

[10] Prakoso A.A., Suprapto J., Subandiyono D. (2020). Influence of Protein and the Level of Energy-Protein Feed Ratio on Growth of Banana Shrimp (*Fenneropenaeus merguiensis* de Man). Int. J. Fish. Aquat. Stud..

[11] Gong F., Zhang N., Guo H., Zhu K., Liu T., Jiang S., Zhang D. (2016). Development and Characterization of 23 Polymorphic Microsatellite Markers for Banana Shrimp *Fenneropenaeus merguiensis*. Conserv. Genet. Resour..

[12] (2016). Determination of Moisture in Food.

[13] (2016). National Standards for Food Safety-Determination of Proteins in Foods.

[14] (2016). National Food Safety Standard-Determination of Crude Fat in Food.

[15] (2016). National Food Safety Standard-Determination of Ash in Food.

[16] (2009). Determination of Total Sugar Content in Edible Fungi.

[17] (2016). National Food Safety Standard-Determination of Amino Acids in Foods by Hydrochloric Acid Hydrolysis.

[18] (2016). National Food Safety Standard-Determination of Fatty Acids in Foods by Gas Chromatography.

[19] Freed D., Aldana R., Weber J.A., Edwards J.S. (2017). The Sentieon Genomics Tools–A Fast and Accurate Solution to Variant Calling from next-Generation Sequence Data. BioRxiv.

[20] Li H. (2013). Aligning Sequence Reads, Clone Sequences and Assembly Contigs with BWA-MEM. arXiv.

[21] Kendig K.I., Baheti S., Bockol M.A., Drucker T.M., Hart S.N., Heldenbrand J.R., Hernaez M., Hudson M.E., Kalmbach M.T., Klee E.W. (2019). Sentieon DNASeq Variant Calling Workflow Demonstrates Strong Computational Performance and Accuracy. Front. Genet..

[22] Chen Y., Chen Y., Shi C., Huang Z., Zhang Y., Li S., Li Y., Ye J., Yu C., Li Z. (2018). SOAPnuke: A MapReduce Acceleration-Supported Software for Integrated Quality Control and Preprocessing of High-Throughput Sequencing Data. Gigascience.

[23] Lin Y., Miao L., Sun C., Jiang W., Zhou Q., Liu B., Ge X. (2022). New Insights on the Effects of In-Pond Raceway Aquaculture System (IRAS) with Dietary Rhubarb Extracts on the Fresh Meat Quality of *Megalobrama amblycephala*. Aquaculture.

[24] Lorenzo I., Serra-Prat M., Yébenes J.C. (2019). The Role of Water Homeostasis in Muscle Function and Frailty: A Review. Nutrients.

[25] Hua K., Cobcroft J.M., Cole A., Condon K., Jerry D.R., Mangott A., Praeger C., Vucko M.J., Zeng C., Zenger K. (2019). The Future of Aquatic Protein: Implications for Protein Sources in Aquaculture Diets. One Earth.

[26] Wang J. (2013). Comparision of Nutritional Composition in Muscle of Penaeus Chinensis, Penaeus Vannamei Boone and *Penaeus japonicuss* Bate. Food Sci. Technol..

[27] Liu Z., Liu Q., Zhang D., Wei S., Sun Q., Xia Q., Shi W., Ji H., Liu S. (2021). Comparison of the Proximate Composition and Nutritional Profile of Byproducts and Edible Parts of Five Species of Shrimp. Foods.

[28] XU XH L., YAN B. (2011). Nutritional Component Analysis and Quality Evaluation of *Penaeus japonicus*. Food Sci..

[29] Fafournoux P., Bruhat A., Jousse C. (2000). Amino Acid Regulation of Gene Expression. Biochem. J..

[30] Hoseini S.M., Reverter M., Gupta S.K., Giri S.S. (2021). Health-Promoting Effects of Amino Acids in Fish. Biotechnological Advances in Aquaculture Health Management.

[31] Duran B.O.S., Zanella B.T.T., Perez E.S., Mareco E.A., Blasco J., Dal-Pai-Silva M., Garcia de la Serrana D. (2022). Amino Acids and IGF1 Regulation of Fish Muscle Growth Revealed by Transcriptome and microRNAome Integrative Analyses of Pacu (*Piaractus mesopotamicus*) Myotubes. Int. J. Mol. Sci..

[32] Li G., Li Z., Liu J. (2024). Amino Acids Regulating Skeletal Muscle Metabolism: Mechanisms of Action, Physical Training Dosage Recommendations and Adverse Effects. Nutr. Metab..

[33] Shi L., Hao G., Chen J., Ma S., Weng W. (2020). Nutritional Evaluation of Japanese Abalone (*Haliotis Discus Hannai* Ino) Muscle: Mineral Content, Amino Acid Profile and Protein Digestibility. Food Res. Int..

[34] Tan DeQing T.D., Wang JianWei W.J., Dan ShengGuo D.S. (2004). The Ratio of Flesh to Body and Analysis on Nutritive Composition of Muscle in *Ancherythroculter nigrocauda*. Acta Hydrobiol. Sin..

[35] Tocher D.R. (2010). Fatty Acid Requirements in Ontogeny of Marine and Freshwater Fish. Aquac. Res..

[36] Sargent J., Bell G., McEvoy L., Tocher D., Estevez A. (1999). Recent Developments in the Essential Fatty Acid Nutrition of Fish. Aquaculture.

[37] Yuan J.L., Liu M., Ni M., Mi G.Q., Zhang C., Gu Z.M. (2018). Effects of Different Culture Models on Growth Performances, Morphological Traits and Nutritional Quality in Muscles of *Micropterus salmoides*. Acta Agric. Univ. Jiangxiensis.

[38] Chen J., Liu H. (2020). Nutritional Indices for Assessing Fatty Acids: A Mini-Review. Int. J. Mol. Sci..

[39] Araujo P., Truzzi C., Belghit I., Antonucci M. (2021). The Impact of Seawater Warming on Fatty Acid Composition and Nutritional Quality Indices of *Trematomus bernacchii* from the Antarctic Region. Food Chem..

[40] Simonetto M., Infante M., Sacco R.L., Rundek T., Della-Morte D. (2019). A Novel Anti-Inflammatory Role of Omega-3 PUFAs in Prevention and Treatment of Atherosclerosis and Vascular Cognitive Impairment and Dementia. Nutrients.

[41] Li X., Wang Y., Li H., Jiang X., Ji L., Liu T., Sun Y. (2021). Chemical and Quality Evaluation of Pacific White Shrimp *Litopenaeus vannamei:* Influence of Strains on Flesh Nutrition. Food Sci. Nutr..

[42] Yaz Y.A., Yıldırım N., Yaz Y., Tekin N., İnal M., Şahin F.M. (2019). Role of Oxidative Stress in Pseudoexfoliation Syndrome and Pseudoexfoliation Glaucoma. Turk. J. Ophthalmol..

[43] Zhang LiYing Z.L., Zhao Meng Z.M., Wang YueZhi W.Y. (2012). A Review on Superoxide Dismutases of Hydrobios. Acta Agric. Univ. Jiangxiensis.

[44] Attia H.G., El-Morshedy S.M., Nagy A.M., Ibrahim A.M., Aleraky M., Abdelrahman S.S., Osman S.M., Alasmari S.M., El Raey M.A., Abdelhameed M.F. (2024). Citrus Clementine Peel Essential Oil Ameliorates Potassium Dichromate-Induced Lung Injury: Insights into the PI3K/AKT Pathway. Metabolites.

[45] Ren Z., Fan Y., Zhang Z., Chen C., Chen C., Wang X., Deng J., Peng G., Hu Y., Cao S. (2018). Sodium Selenite Inhibits Deoxynivalenol-Induced Injury in GPX1-Knockdown Porcine Splenic Lymphocytes in Culture. Sci. Rep..

[46] Silvestrini A., Meucci E., Ricerca B.M., Mancini A. (2023). Total Antioxidant Capacity: Biochemical Aspects and Clinical Significance. Int. J. Mol. Sci..

[47] Carmo De Carvalho E Martins M.D., Martins, Da Silva Santos Oliveira A.S., Da Silva L.A.A., Primo M.G.S., De Carvalho Lira V.B., Patel V.B., Preedy V.R. (2022). Biological Indicators of Oxidative Stress [Malondialdehyde, Catalase, Glutathione Peroxidase, and Superoxide Dismutase] and Their Application in Nutrition. Biomarkers in Nutrition.

[48] Tan S.H., Deng X.Y., Jiang W.M., He F.L. (2007). Effects of High Level Chromium on Antioxidant Enzyme System in Gill and Hepatopancreas of Procambarus Clarkii. J. Agro-Env. Sci..

[49] Tang Q.-Y., Xie J.-H., Xia Z.-L., Cai M.-Y., Wu Y.-M., Bai L.-H., Du H.-K., Li J.-F., Yang G.-L. (2020). Genetic Diversity of the Breeding Populations of Giant Freshwater Prawn *Macrobrachium rosenbergii*. Acta Hydrobiol. Sin..

[50] O’Connell M., Wright J.M. (1997). Microsatellite DNA in Fishes. Rev. Fish Biol. Fish..

[51] Jian-lin L.I., Hong-xia L.I., Yong-kai T., Ju-hua Y.U., Fan Y.U. (2015). Genetic Difference Analysis of Two Genetically Improved Farmed Tilapia Populations by Using Microsatellite Marker. J. South. Agric..

[52] Serrote C.M.L., Reiniger L.R.S., Silva K.B., dos Santos Rabaiolli S.M., Stefanel C.M. (2020). Determining the Polymorphism Information Content of a Molecular Marker. Gene.

[53] Matukumalli L.K., Lawley C.T., Schnabel R.D., Taylor J.F., Allan M.F., Heaton M.P., O’Connell J., Moore S.S., Smith T.P., Sonstegard T.S. (2009). Development and Characterization of a High Density SNP Genotyping Assay for Cattle. PLoS ONE.

[54] Luo W., Luo C., Wang M., Guo L., Chen X., Li Z., Zheng M., Folaniyi B.S., Luo W., Shu D. (2020). Genome Diversity of Chinese Indigenous Chicken and the Selective Signatures in Chinese Gamecock Chicken. Sci. Rep..

[55] Holsinger K.E., Weir B.S. (2009). Genetics in Geographically Structured Populations: Defining, Estimating and Interpreting F ST. Nat. Rev. Genet..

